# Insights into Fisheries Management Practices: Using the Theory of Planned Behavior to Explain Fish Stocking among a Sample of Swiss Anglers

**DOI:** 10.1371/journal.pone.0115360

**Published:** 2014-12-16

**Authors:** Eike von Lindern, Hans-Joachim Mosler

**Affiliations:** Environmental and Health Psychology, Eawag, Dübendorf, Switzerland; Leibniz Center for Tropical Marine Ecology, Germany

## Abstract

Using inadequate management tools often threatens the natural environment. This study focuses on the example of Swiss recreational fishermen (hereafter called “anglers”) as recreational fisheries management stakeholders. In recreational fisheries, fish stocking conducted by anglers has been identified as one important factor associated with declining fish catches. We therefore aimed to a) gain insights into why anglers want to maintain fish stocking and b) identify entry points for interventions to promote more pro-ecological management practices. Results (N = 349) showed that the majority of anglers think very uncritically about stocking and that they frequently engage in it. We conclude that outcome expectancies and beliefs about risks, in combination with a lack of stocking success controls are the main reasons that anglers retain stocking measures. We suggest that providing anglers with direct experience and feedback about stocking success is suitable to change their intentions regarding stocking and their actual stocking behavior, and thus, to promote more pro-ecological management methods. From a more general perspective, the results of this study are likely to help improve pro-ecological ecosystem management in other domains where problems similar to those in recreational fisheries management might exist.

## Introduction

Human behavior and management decisions are relevant for managing natural resources [1]. Concerning the management of stream and river ecosystems in the frame of recreational fisheries, research has shown that serious changes have occurred in recent decades. The decline in inland fish catches has become a topical issue in many countries [2]–[4]. In Switzerland, catches of brown trout (*Salmo trutta*, hereafter called “trout”) have decreased by more than 60% since 1980 [2]. Three most likely reasons for declining fish catches, namely the habitat situation of running waters (e.g., morphology and water quality), a parasitic fish disease (e.g., proliferative kidney disease, for details see [5]), and improper fisheries management were identified as major impact factors (e.g., [2]).

Consequently, in the present study, improper fisheries management will be focused on as a direct, human-caused impact on stream and river ecosystems. Recreational fishermen (hereafter called “anglers”) dominate the inland fisheries sector in many industrialized countries [3], [6] and can be considered main stakeholders and key players in fisheries management [3], [7]. Anglers are direct users of stream and river ecosystems and are at the same time involved in ecosystem management. They are the sole fisheries users in Swiss running waters and they actively participate in fisheries management activities.

On the one hand, anglers can contribute to fisheries conservation [8], but they also have the potential to threaten stream and river ecosystems and biodiversity through exploitation and inadequate or maladjusted management decisions [8]–[10]. One of the most widespread management tools is “fish stocking”, which is very popular among anglers [4], [7], [9], [11]. This can be defined as the intentional release of large numbers of fish into a body of water. Anglers consider stocking to be the ultimate and immediate solution for declining fish stocks and catches [4]. Among the main motives for stocking are mitigation of human-caused habitat perturbations (e.g., lack of spawning sites), restoration (e.g., stock recoveries after fish-kills or habitat improvements), conservation (e.g., retaining populations threatened by extinction), and harvest enhancement [6], [12]–[14]. In Swiss running waters, trout are the fish species most commonly stocked (and caught) by anglers [2]. Trout for stocking are usually derived from adults that have been caught from the wild or held in hatcheries. Their offspring are reared to a certain age in hatcheries or (semi-) natural rearing streams and ponds.

### Stocking as an example of inadequate ecosystem management

The number of stocked fish is remarkable. Cooke and Cowx [9] estimate that approximately 40 billion fish are stocked annually in European fresh waters and point out that a similar stocking scale is common around the world. In Switzerland, nearly 660 million fish were stocked in 2004, and stocking was conducted in 88% of almost 3000 Swiss stream and river sections listed in the national fisheries statistics [15]. Management of running waters in Switzerland officially falls under the department for hunting and fisheries of each Swiss canton’s administration [16]. With regard to the “Bundesgesetz über die Fischerei” [17], cantonal fisheries inspectors are in charge of stocking activities in their canton. However, the fisheries inspectors work closely together with angling clubs. This means that stocking is mostly performed by members of the angling clubs.

From a biological and psychological perspective, current stocking practice can be understood as an example of inadequate ecosystem management. Stocking and its impact on stream and river ecosystems, as well as its success or failure, have been pursued mostly uncritically in the past [3], [18] but have recently been increasingly questioned by fisheries biologists and ecologists [9], [19]. In particular, fish stocking is considered a potential threat to fish conservation and the sustainability of indigenous fish stocks. Stocking can harm native fish stocks through increased competition (between and within fish stocks), loss of genetic distinctiveness (e.g., through hybridization), and through the spread of diseases and/or parasites [9], [10]. Additionally, stocking success controls are rarely conducted and the contribution of stocking to the overall size of trout stocks is likely to be overestimated by anglers [2]. Furthermore, despite of lack of success or even proven failure, Swiss anglers intend to continue stocking or even increase it [20]. In this sense, fish stocking conducted by anglers can be defined as a case of inadequate ecosystem management.

Furthermore, peer pressure by anglers is important for management decisions in fisheries [11], [21], [22]. Anglers think of peers as a reliable and trustworthy source of experience and knowledge [23], whereas other sources, for example, scientists or agency officials, are not necessarily rated as trustworthy [24] (cited after [23]). For these reasons in combination with the anglers’ generally positive attitudes toward stocking and the traditional character of stocking in Switzerland, stocking as a management tool has become a social norm for individual anglers. The normative character of stocking may, of course, vary depending on how strongly an angler is integrated into a social network of peers (e.g., is a member of a fishing club).

In summation, anglers in Switzerland are directly involved in stocking activities and they favor stocking as a management tool. In general, anglers intend to continue with stocking or even increase stocking activities. They tend to trust peers more than other sources of information concerning fisheries-related experiences and knowledge. On the other hand, research in fisheries management and ecology indicates that stocking benefits are likely overestimated, and stocking success controls are rarely done. Stocking can also be considered a possible threat to fish conservation through increased competition, loss of genetic integrity, and the spread of diseases and parasites.

### Aim of study

The framework of anglers’ stocking activities provides an excellent research setting for generating a deeper understanding of inadequate ecosystem management practices. Why do anglers favor stocking as a management tool, and why do they intend to maintain or even increase stocking efforts despite the questions raised by scientists and experts regarding stocking outcomes? How might the anglers’ convictions concerning stocking be changed? To address these questions, it will be necessary to assess the anglers’ beliefs about the risks associated with stocking and the anglers’ outcome expectancies regarding stocking in combination with their attitudes toward stocking and intention to engage in (future) stocking activities. With this approach, we can determine the extent to which the above-mentioned controversy is represented by the anglers’ beliefs about, and attitudes toward, stocking and whether this representation can be associated with anglers’ engagement in stocking-related activities.

### Theory of planned behavior as theoretical framework

In the present study, we apply the theory of planned behavior (“TPB”, [25], [26]) to assess the anglers’ views and beliefs concerning stocking from a psychological perspective. More specifically, we focus on the influence of the anglers’ outcome expectancies and their beliefs about stocking-related risks on their attitudes toward stocking and their intention to engage in stocking. According to the stocking controversy summarized above, we assume that if anglers really think mostly positively about stocking, see it as a panacea for declining fish catches, and likely overestimate its impact, as suggested by, for example, Welcomme and Bartley [3] or Burkhardt-Holm et al. [2], then this should also be reflected by high outcome expectancies concerning stocking measures as well as by a tendency to neglect stocking-related risks. Thus, it will lead to retaining stocking, though it may be an improper fisheries management tool. Additionally, we assume that anglers in Switzerland have the chance to participate in stocking, or at least have the opportunity to engage in stocking-related activities, because, in Switzerland, stocking is traditional and mostly conducted by members of fishing clubs.

With this said, the TPB [25], [26] provides a well-suited theoretical framework to deepen our understanding of the anglers’ management behavior. The theory has been widely used to explain behavior in different domains such as environmental activism (e.g., [27], [28]), energy consumption behavior (e.g., [29]), and management of natural resources (e.g., [30]). A basic assumption of the TPB is that performing a certain behavior depends on the intention to perform that behavior. The intention, in turn, is influenced by perceived norms, by attitudes toward the behavior (or its expected outcomes), and by the perceived behavioral control [26]. Within the TPB framework norms refer to an individual’s beliefs about what one should do, either through personal values, the values of significant others, or through values held by a specific group or society. Attitudes reflect the assessment of whether a specific behavior or associated outcome is seen as bad and unfavorable or good and favorable. Perceived behavioral control represents how strongly an individual is convinced that he/she has the skills, resources, and capabilities to actually perform the behavior in question.

**Figure 1:**
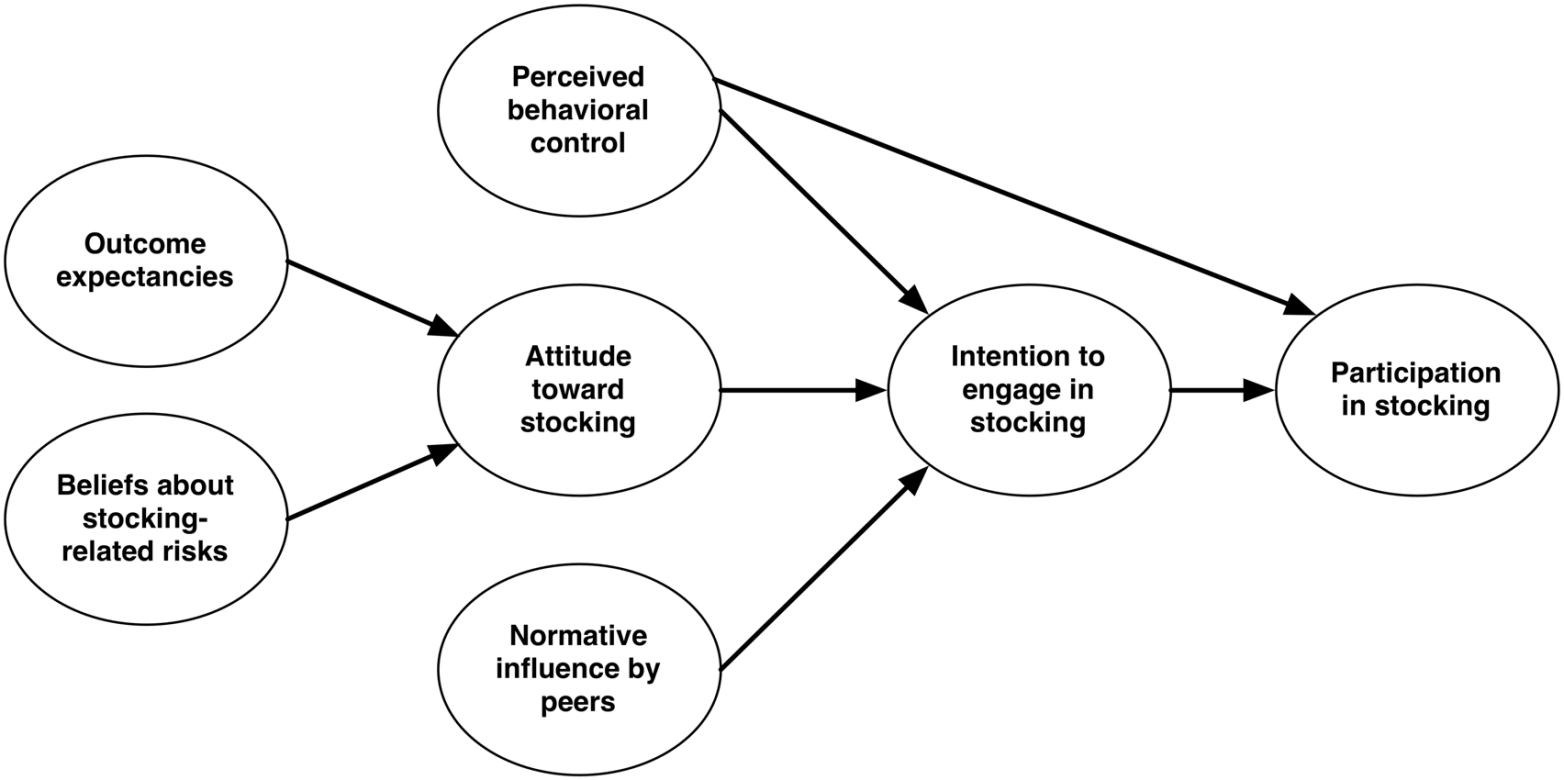
Conceptual model of constructs influencing an angler’s intention to engagement in stocking-related activities.

Beliefs about a behavior strongly influence the attitude toward the respective behavior, among other so-called background factors [26]. As depicted in Fig. 1, we were particularly interested in how beliefs about stocking-related risks and outcome expectancies influence the anglers’ attitudes toward stocking and how their attitudes, peer pressure as a social norm, and perceived behavioral control influence their intentions and engagement regarding stocking.

## Methods

### Ethics statement

According to the initial review of the Ethical Review Board of the Department of Psychology at the University Zürich, Switzerland (http://www.phil.uzh.ch/forschung/ethik.html) the research did not contain any critical points concerning ethics in research. This means in detail that the present study followed the ethical guidelines of the American Psychological Association, and the survey procedure conformed to the Declaration of Helsinki (see also description of the procedure, below). Thus, no additional ethics approval was required for this survey study.

### Procedure, informed consent, and statistical analysis

We conducted a mail survey of 1669 Swiss anglers. Postal addresses for the survey were retrieved through institutional and cantonal fishing databases. We additionally distributed a link to an online version of the questionnaire (with identical wording and layout) through fisheries-related websites. Completing the questionnaire was rather demanding and took approximately 90 minutes as it contained additional items and topics as part of a larger survey on fisheries and related domains aside from measures used for the present study. However, a pretest among a random subsample (n = 20) drawn from the same database of anglers rendered its length and time effort feasible.

The survey included a cover page with written information about the research project. It contained information about the overall research topic, the purpose of the study, methods used and information about the entity responsible for the research. It additionally stated that participation was completely voluntary and that participation could be ended anytime the respondents wished without giving reasons. Participants were told that all data would be processed for scientific analyses and publication only and that any personal information would be anonymized and would never be given to any third party. Informed consent was obtained by stating that participants should only fill in the questionnaire and return it if they understood the information and agreed with it. As an incentive for participation, we offered a short report on the main findings (comprehensible for laymen).

The response rate was 25% and thus slightly lower than expected. We had to exclude data from 69 individuals who stated that they were not members of a club. This exclusion criterion applied because conducting stocking in Switzerland is only allowed for members of fishing clubs under agreement of cantonal fisheries inspectors. Our target group for this survey, therefore, only consisted of those anglers who were members of a fishing club at the time when we conducted the study. Thus, our final sample comprises data from N = 349 Swiss anglers. All returned questionnaires contained consistent answers, so we did not need to exclude additional cases. Missing data were treated with full information maximum likelihood estimation (FIML; [31]). FIML procedures generate more reliable results than, for example, listwise or pairwise case deletion [32], [33] and are considered to have the equivalent power of multiple imputation procedures [34]. We approached the research question by employing a structural equation model (SEM). SEM approaches provide several advantages over, for example, regression analysis. The SEM approach unifies several multivariate methods into one analytical framework, allows the testing of complex hypotheses among latent variables, and provides information about the effect of latent variables on each other and on manifest variables (e.g., [35], [36]). In order to answer our research question we formulated a SEM that represents assumptions stated by the TPB (see conceptual model, Fig. 1). In the SEM, we employed several manifest variables for measuring each underlying latent TPB construct. Details on the measurements are provided in the measurements section below. All analyses were conducted with Mplus Version 6.1 [35] and R version 3.1.1 for MAC. Structural equation modeling was mainly performed with the R package lavaan [36]. Non-normality of data was addressed by calculating robust standard errors. Please note that we collected data in a cross-sectional study design only. Therefore, our analysis of the data does not allow interpretation in a strict causal sense. All relevant data are within the paper and its Supporting Information files.

### Participants

Almost all respondents were male (99%), and their mean age was 53.4 (*SD* = 13.8) years. They can be considered experienced anglers with a mean angling experience of 38.3 (*SD* = 15.3) years. All anglers were active members of fishing clubs, and 87% stated that they had already engaged in stocking-related activities in the past. Their educational level was rather low: the highest education achieved by the majority was to graduate from vocational school (1% no school degree; 4% primary school; 52% vocational school; 33% higher professional training; 10% technical college/university).

Comparing the socio-demographics to a nationwide study conducted by Schwärzel-Klingenstein, Lüthi, and Weiss [20] with N = 1287 respondents, we can conclude that the respondents in our sample are comparable in their age structure (60% were between 30 to 60 years old). However, with a share of 99% instead of 96%, a slightly higher percentage of male anglers responded to our survey. Also, other socio-demographic indicators, like angling experience, were comparably high in both studies. Though this comparison indicates that the sampling procedure we used seemed to reach those anglers who are also willing to respond to other questionnaire studies, we acknowledge that this comparison does not necessarily make strong claims for our sample being representative of the total population of Swiss anglers. However, with regard to the socio-demographic characteristics, we argue that anglers belonging to our sample represent the population of those Swiss anglers who can be reached by survey methods. We are aware that other studies may also be impacted by some kind of bias, meaning that generalizations about the whole population of anglers should not be made without appropriate caution or without providing further supporting arguments.

### Measures

*Attitude toward stocking* was assessed by three items. The wording was “In general, I think stocking measures are…” (Att_1), “I think stocking in (near-) natural rivers is…” (Att_2), and “I think stocking in degraded rivers is…” (Att_3). All items could be answered on a 5-point Likert scale ranging from 1 (*very bad*) to 5 (*very good*). These items represent whether an angler thinks of stocking positively or negatively. We differentiated between (near-) natural and degraded streams and rivers to cover the whole range of environmental states of streams and rivers in Switzerland. The mean scale score was 10.37 (*SD* = 2.49) and ranged from 3 to 15. The higher the scale score, the more positively an angler thinks about stocking. The Cronbach’s alpha of.72 suggests satisfactorily high reliability.

*Intention to engage in stocking* was assessed with two items. They were worded “How strongly are you willing to participate actively in stocking activities?” (Int_1) and “How strongly do you intend to engage in stocking activities?” (Int_2). The answer scale for both items ranged from 1 (*not at all*) to 5 (*very strongly*) on a 5-point Likert scale. The mean scale score was 8.12 (*SD* = 2.44), ranging from 2 to 10. A higher score reflects a stronger intention to participate in stocking activities. Reliability analysis resulted in a Cronbach’s alpha of.72, which can be interpreted as satisfactorily high.

*Outcome expectancies* were measured with a total of two items. The first item concerned how far the believed share of stocked trout in the anglers’ average catch was perceived as indicating the success or failure of stocking measures. The angler first had to provide his/her guess of the share of stocked trout in the catch, and then he/she had to follow up on this assessment by completing this sentence: “This share suggests that stocking measures were…” (OE_1). Options for completing the sentence were provided as a 4-point rating scale with the verbal anchors *not at all successful* **(**1), *rather not successful* (2), *rather successful* (3), and *very successful* (4). The second item was worded as follows: “I think that stocking measures of which I personally know of were…” (OE_2) and could be answered on the same scale as OE_1. The mean scale score was 5.56 (*SD* = 1.39). The higher the score, the higher the outcome expectancy an angler had with regard to stocking. Cronbach’s alpha value of.81 suggests a high reliability for this scale.

*Perceived behavioral control* was represented by a single item measure. The wording was “It is possible for me to participate in stocking-related activities in my fishing club” (PBC), with the answer scale ranging from 1 (*absolutely disagree*) to 5 (*absolutely agree*). This item referred to the odds that a given angler has to participate in stocking. The mean item score was 3.85 (*SD* = 1.33). A higher score reflects stronger perceived behavioral control concerning participating in activities related to stocking.

*Beliefs about stocking-related risks* were assessed by a multiple-choice item (Risks). The wording was “From my point of view, stocking can in the worst case…” followed by the answer options “be not successful”, “increase intraspecific competition”, “increase interspecific competition”, “introduce and/or spread diseases and parasites”, “increase number of (avian) predators”, “lead to hybridization between locally adapted and stocked trout”, “Stocking is always positive”, and “I do not know”. We derived the answer options for potential risks associated with stocking from both literature (intraspecific competition: [37]; diseases and parasites: [38], [39]; hybridization: [40], [41]) and answers frequently obtained in a preliminary qualitative interview study (lack of success; avian predators; interspecific competition). Although lack of success poses no real risk for the ecosystem, it is nevertheless a risk with regard to finances and effort. “Stocking is always positive” was provided as an answer option given the general faith in stocking described in anglers [4], [7], [11]. “I do not know” was provided because stocking has often been pursued uncritically, with little scientific evaluation of its success or failure [3], [18]. An angler could therefore not have been confronted with or thought of possible stocking risks and should not be forced to mark anything else in that case.

We calculated a score based on the number of risks an angler associates with stocking. This initial risk score of “0” was increased by +1 for every risk an angler marked, meaning that the score could range from “0” to “6”. Thus, the higher values indicate the association stocking with more risks. The answer option “I do not know” did not contribute to the score because it represents an angler’s uncertainty rather than his/her beliefs regarding stocking-related risks. If an angler checked the option “stocking is always positive”, the sum score was fixed at “0”, no matter whether other additional risks were mentioned.

*Normative influence by peers* was measured with three items, which represent the (self-reported) influence of the fishing clubs’ or peers’ attitudes toward stocking on the respondents’ own attitudes, the importance of belonging to the club or peer group, and a judgment of how much credibility an individual angler assigns to his/her fellow club members regarding fish stocking. The items were worded thus: “With regard to fish stocking, I consider knowledge and experiences of my fellow club members as more credible compared to any other source.” (Norm_1), “Belonging to the fishing club is for me…” (Norm_2), and “The influence of my angling club’s views on stocking on my attitude towards stocking is…” (Norm_3). Norm_1 could be answered on a 5-point Likert scale ranging from 1 (*not at all agree*) to 5 (*totally agree*); the answer scale for Norm_2 ranged from 1 (*unimportant*) to 4 (*important*); and for Norm_3 the scale ranged from 1 (*(almost) negligible*) to 5 (*very strong*). The mean scale score was 9.92 (*SD* = 2.29) with a minimum of 3 and a maximum of 14. Higher scores reflect stronger influence by peers from the fishing club with regard to stocking. Cronbach’s alpha was.42, which suggests only a low reliability of this measure. This means that the results related to normative influences by peers should be interpreted with caution.

*Stocking behavior* was measured by a total of five items. The items asked the participants how frequently they participated in harvesting fish for stocking (BEH_1), electro-fishing (BEH_2), activities associated with rearing trout for stocking (BEH_3), fish stocking (BEH_4), and catching of spawners (BEH_5). All items could be answered on a 5-point scale, ranging from 1 (*never*) to 5 (*always*). The mean scale score was 14.1 (*SD* = 5.72) with a minimum of 5 and a maximum of 25. A higher score reflects higher frequencies of participation in stocking-related activities in the fishing club. The Cronbach’s alpha of.84 suggests good reliability for this measure. Stocking behavior thus reflects the self-reported assessment of past and current behavior that can be associated with stocking. It was not possible to actually assess future stocking behavior because of the cross-sectional study design. From how far in the past behavior can serve as a proxy for future behavior is particularly a question of the stability of behavioral patterns and circumstances. A large body of literature concludes that past behavior is a very strong predictor for future behavior, especially if the circumstances remain relatively stable and are not changed, for example, by interventions (e.g., [42]–[44]). This is likely the case with fish stocking conducted by fishing clubs because members of the fishing clubs spend effort in terms of money and working hours in building and maintaining infrastructure needed for conducting stocking. Thus, the environmental circumstances are relatively stable, and stocking is also regarded as a traditional management tool, which dates back to the 1870s ([12], [45]). We therefore argue that the frequency of engagement in past and actual stocking-related activities can be defined as a valid proxy for engaging in future stocking behavior.

All items were originally formulated in German.

## Results

The descriptive results revealed that the surveyed anglers had, on average, a positive attitude toward stocking, a strong intention to engage in stocking, expected stocking to be rather successful, and reported a rather high behavioral control concerning stocking-related activities. Furthermore, they mentioned 2.4 out of 6 risks that can be associated with stocking, and they acknowledged that their peers’ opinions had a quite strong influence on their own opinions. The surveyed anglers engaged quite frequently, on average, in activities that are associated with stocking behavior (see Table 1).

**Table 1:** Means (*M*), standard deviations (*SD*), range, and correlation matrix for the variables in the analysis.

Construct	Variable	*M* (*SD*)	Range	Correlations																			
Attitudes toward stocking	Att_1	3.65 (1.24)	1–5	(1)																			
	Att_2	3.21 (1.26)	1–5	.57**	(2)																		
	Att_3	3.57 (1.23)	1–5	.12*	−.18**	(3)																	
Intention to do stocking	Int_1	3.97 (1.40)	1–5	.29**	.17**	.02	(4)																
	Int_2	4.13 (1.39)	1–5	.25**	.14*	.08	.56**	(5)															
Stocking behavior	Beh_1	3.42 (1.44)	1–5	.07	**−**.03	**−**.07	.46**	.27**	(6)														
	Beh_2	2.98 (1.48)	1–5	**−**.03	−.15*	.02	.34**	.18**	.75**	(7)													
	Beh_3	2.66 (1.60)	1–5	.08	.08	**−**.06	.42**	.32**	.50**	.42**	(8)												
	Beh_4	3.55 (1.39)	1–5	.14*	.03	**−**.01	.60**	.37**	.62**	.52**	.50**	(9)											
	Beh_5	2.32 (1.44)	1–5	.05	**−**.04	**−**.01	.38**	.23**	.46**	.50**	.56**	.41**	(10)										
Perceived behavioral control	PBC	3.85 (1.33)	1–5	.01	.06	−.15**	.31**	.16**	.37**	.33**	.33**	.34**	.25**	(11)									
Beliefs about stocking risks	Risks	2.41 (1.68)	0–6	−.41**	−.50**	.02	−.11*	**−**.07	**−**.03	.04	**−**.09	**−**.04	.05	.01	(12)								
Normative influence by peers	Norm_1	3.26 (1.12)	1–5	.22**	.29**	**−**.07	.06	**−**.05	.00	**−**.05	.01	.03	**−**.07	.18**	−.34**	(13)							
	Norm_2	3.52 (.72)	1–4	.01	.02	.00	.18**	.13*	.29**	.18**	.22**	.23**	.08	.19**	**−**.03	.12*	(14)						
	Norm_3	3.16 (1.42)	1–5	.20**	.16**	**−**.10	.24**	.20**	.22**	.19**	.18**	.23**	.20**	.34**	−.17**	.24**	.27**	(15)					
Outcome expectancies	OE_1	2.76 (.76)	1–4	.35**	.30**	.01	.03	.05	.06	.01	.15*	.07	.13	.03	−.17**	.06	.04	.09	(16)				
	OE_2	2.77 (.73)	1–4	.44**	.32**	**−**.07	.18**	.18**	.14*	**−**.02	.19**	.15*	.12	**−**.01	−.19**	.09	.05	.12*	.67**	(17)			
Demographics	Age (years)	53.37 (13.83)	16–85	.12*	.19**	**−**.03	**−**.00	.01	.05	**−**.00	.12*	.10	**−**.03	**−**.01	−.21**	.10	.14*	.08	.03	.00	(18)		
	Education^a^	3.88 (1.21)	1–5	**−**.05	−.17**	.08	**−**.10	**−**.09	−.18**	**−**.04	−.19**	**−**.08	**−**.03	**−**.07	.12*	−.19**	−.19**	−.13*	**−**.07	**−**.06	**−**.03	(19)	
	Fishing exp. (years)	38.28 (15.28)	2–80	.07	.07	.03	**−**.02	**−**.02	**−**.00	.04	.13*	.06	.02	**−**.04	**−**.05	**−**.01	.08	**−**.03	**−**.01	**−**.07	.70**	.02	(20)
	Gender^b^	1.99 (.11)	1–2	.08	.02	.02	.00	**−**.04	.02	**−**.05	.01	.05	.03	**−**.08	.01	.00	**−**.04	**−**.06	**−**.03	.09	.12	**−**.01	.13*

*Note*. All N = 349; * = p<0.05; ** = p<0.01.

a Codes for education: 1 = no degree, 2 = primary school, 3 = vocational school, 4 = higher professional training, 5 = technical college/university; higher numbers indicate higher level of education.

b Codes for gender: 1 = female, 2 = male. Please note that almost all participants were male, therefore gender was excluded from all further analyses.

The correlations between the variables in the analysis and demographic variables indicated that there were mainly substantial and systematical associations between items within the same constructs, for instance, within stocking-related behaviors. However, the correlation matrix also indicated that, in general, a more positive attitude toward stocking was related to a higher intention to engage in stocking, higher outcome expectancies, association of fewer risks with stocking, and a stronger normative influence by peers. Perceived behavioral control was only marginally and negatively associated with an anglers’ attitude toward stocking. The intention to engage in stocking correlated significantly with measures for attitude, outcome expectancies, perceived behavioral control, normative influence by peers, and engagement in stocking behavior, but only slightly with beliefs about stocking risks. Perceived behavioral control was additionally positively associated with normative influence by peers but not correlated with outcome expectancies. Overall, we found mostly moderate correlations between all variables, whereas the strongest correlations were mainly within the measurements of each latent dimension (see Table 1 for details).

Results from the structural equation modeling (SEM) on the basis of TPB ([26]; see also Fig. 1, above) indicated that the empirical data fit the model assumptions moderately well. The standard fit indices available for such models were as follows: RMSEA = 0.05, 90%-CI [0.04, 0.06]; CFI = 0.93; TLI = 0.91, SRMR = 0.06; Chi2/dF = 2.02. Details on model fit criteria are discussed in, for example, Hu and Bentler [46] or Geiser [47].

**Figure 2:**
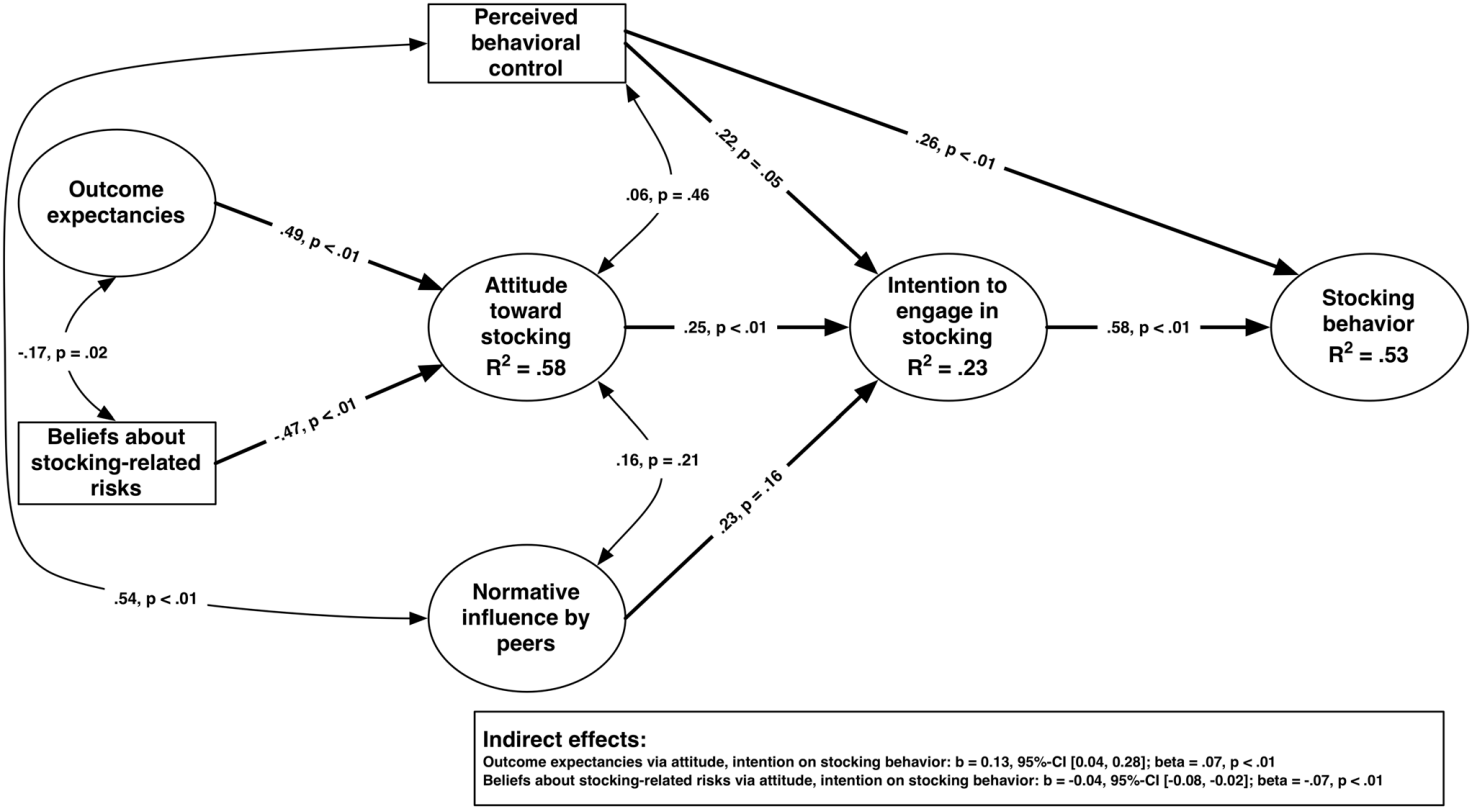
Structural equation model of latent constructs influencing an angler’s engagement in stocking-related activities. All path coefficients are standardized values; error terms are omitted due to reducing complexity of the figure.

The underlying model assumption of the TPB explained a huge amount of variance concerning the anglers’ attitudes toward stocking (R^2^ = 0.58), their intention to engage in stocking (R^2^ = 0.23), and their engagement in stocking-related behavior (R^2^ = 0.53) (Fig. 2).

Besides the amount of explained variance for the latent variables attitude toward stocking, intention to engage in stocking, and stocking behavior, the results obtained from the SEM indicated that perceived behavioral control had a significant influence on engaging in stocking behavior (b = 0.21, 95%-CI [0.12, 0.29]; beta = .26) and on the intention to engage in stocking (b = 0.20, 95%-CI [0.00, 0.41]; beta = .22). The normative influence by peers did not turn out to impact the anglers’ intentions significantly (b = .34, 95%-CI [−0.14, 0.81]; beta = .23). However, it correlated strongly with perceived behavioral control (r = .54, p<.01). Regarding the attitudes toward stocking, the SEM indicated that it was positively influenced by outcome expectancies (b = 0.80, 95%-CI [0.52, 1.14]; beta = .49) and negatively by beliefs about stocking-related risks (b = −0.27, 95%-CI [−0.33, −0.20]; beta = −.47). This means that having high outcome expectancies and believing that stocking is only related to a few risks (if to any at all) will lead to a more positive attitude toward stocking, which in turn contributes significantly to a stronger intention to engage in stocking and eventually to more frequent engagement in stocking behavior. The latter assumption was tested by calculating the indirect effects from outcome expectancies and beliefs about risks, respectively, on stocking behavior. The indirect influence paths from outcome expectancies mediated via attitude and intention on behavior was highly significant and positive (b = 0.13, 95%-CI [0.04, 0.28]; beta = .07). Thus, for instance, having high outcome expectancies is not only associated with a more positive attitude toward stocking but also with a more frequent engagement in stocking behavior. On the contrary, holding more beliefs about stocking risks negatively impacted the engagement in stocking behavior: the indirect path from beliefs about stocking risks via attitude and intention on behavior indicated a significant and equally strong, but negative, indirect effect (b = −0.04, 95%-CI [−0.08, −0.02]; beta = −.07).

## Discussion

The descriptive results indicate that anglers in our sample are frequently involved in stocking activities in running waters as 87% stated that they have at least once participated in stocking-related activities and the items on frequencies of being involved in stocking-related activities received relatively high mean scores (see also Table 1). By this, the results agree with statements by Welcomme and Bartley [3] and Arlinghaus et al. [6], who point out that anglers are key players in management. The high values for the measure of perceived behavioral control suggest that participation in stocking-related activities is well-established among anglers. The significant correlations with normative influences by peers hint that management decisions do not represent individual management preferences but club- or group-specific ones. From the perspective of the controversy about stocking described above (e.g., [4], [12], [18], [19]), this means that an anglers’ fishing club or other relevant peer group also has to be taken into account when assessing management decisions and processes.

The results further imply that stocking is very popular among our sample of Swiss anglers as their mean attitude toward stocking was very positive (see Table 1). However, the surveyed anglers seem to differentiate between stocking measures in dependence of the environmental state of the stream or river. Looking at the attitude toward stocking in degraded rivers and in near-natural rivers, it is striking that these attitude items correlate negatively (r = −.18, p<.01). This means that anglers who think positively of stocking in degraded rivers tend to rate stocking in near-natural rivers as being rather negative. Anglers seem to discern the feasibility of stocking with regard to the state of the environment. This makes particular sense from a biological perspective: trout need a natural or near-natural habitat to maintain self-sustaining populations [2]. A habitat suitable for salmonids (salmon and trout) is a complex and interactive mixture of water quality, quantity, and physical structure. If any component is inadequate or degraded by human activities or construction, salmonid productivity will decline [48]. In cases where natural reproduction is low due to anthropogenic impairments of, for example, the spawning or rearing habitat, stocking has the potential to increase population abundance [49]. However, in (near-natural) rivers and streams, conditions for natural reproduction are favorable, and the number of recruits might correspond to the carrying capacity of the habitat. In that case, stocking is unnecessary and, if conducted anyway, likely to be unsuccessful or even harmful. The results suggest that anglers also judge stocking measures according to this explanation from fisheries biology or have a similar reasoning. This finding further suggests that some anglers are aware of biological factors that hinder or foster natural trout reproduction and that they do not rate stocking positively under any condition. Moreover, this means that the stocking controversy is, to some degree, present in the anglers’ attitudes toward stocking. On the contrary, the surveyed anglers mentioned, on average, only 2.4 risks that they associated with stocking. The average number of risks mentioned could be considered rather low, especially because most surveyed anglers have already participated in stocking-related activities: 86% have participated in stocking at least once, and 76% reported that they were involved in stocking more or less regularly. Additionally, the average length of fishing experience of 38.3 years (*SD* = 15.3 years), in combination with being members of clubs that are actively involved in stocking, suggests that the anglers have likely heard of most, if not all, of the possible risks mentioned. However, the average of 2.4 risks mentioned indicates that only a relatively small share of the possible risks are present in the anglers’ stocking-related belief system. This can be interpreted as anglers being relatively uncritical toward stocking on average. However, we find here a similar pattern as within the attitude items; the more positively an angler rates stocking in general and in near-natural rivers specifically, the fewer risks he/she associates with stocking. Meanwhile, there was no significant correlation between the beliefs about risks and the attitude toward stocking in degraded rivers. This suggests that even if anglers have contrasting attitudes towards stocking in degraded and near-natural rivers, they are likely to underestimate the potential risks associated with stocking, especially in near-natural rivers.

The rather high outcome expectancies mentioned by the majority of the anglers in our sample provides further evidence for the statement by Burkhardt-Holm et al. [2] that anglers likely overestimate the impact of stocking measures on the overall size of trout stocks. This also reflects the generally positive attitudes and strong intentions of anglers concerning stocking in our sample.

The results from the structural equation model suggest that the TPB provides a sound framework for explaining the anglers’ intentions to engage in stocking. Participating in stocking-related activities was significantly associated with the intention to participate in stocking. The intention, in turn, could be explained by attitudes toward stocking and the perceived behavioral control. The attitude toward stocking could be associated with outcome expectancies and beliefs about stocking risks. The SEM also revealed that outcome expectancies and beliefs about stocking risks impact the anglers’ involvement in stocking, mediated through attitude and intention. Thus, we can conclude that it is decisive for the anglers’ participation in stocking-related behavior what he/she thinks and believes regarding stocking outcomes and risks related to stocking.

We were surprised that the path coefficient from normative influence by peers to intention to engage in stocking was quite weak and statistically insignificant. Findings and results from, for instance, Arlinghaus [50], Jackson et al. [11], Deadlow et al. [21], and van Poorten et al. [22] indicated that pressure by peers would significantly impact the intention to engage in stocking; thus, we expected similar results. One explanation for this unexpected finding might be the strong positive correlation of normative influence with perceived behavioral control. This means that if an angler perceived low normative pressure by peers, he/she was also less likely to have the opportunity to participate in stocking at all. Or, in other words, if it was not possible for a member of the club to engage in stocking, normative influence by peers regarding stocking became unimportant. Furthermore, the measure we used for normative influences by peers turned out to be rather unreliable, as it only received a Cronbach’s alpha value of.42. Accordingly, the factor loadings obtained from the measurement model (as part of the SEM) also suggested that the used items did not reflect the latent construct very well. To conclude, the limitation of having a rather weak and unreliable measure for normative influences by peers is likely to have contributed to the non-significance of the path coefficient. This means that the normative influence may be underestimated in our model; therefore, those results that are linked to normative influences should especially be interpreted with caution. However, the influence of perceived behavioral control was as expected: the more an angler was convinced that he/she was capable of participating in stocking-related work, the higher his/her intention was and the more frequently he/she was involved in stocking-related behavior.

Linking the results of the present study to the discussion about stocking, a promising approach emerges that is suitable for promoting a more pro-ecological ecosystem management. Although past behavior can hardly be changed by intervention, attitudes and beliefs can. Outcome expectancies and risk beliefs provide especially good entry points for interventions: we suggest that stocking success controls, which are seldom conducted, should target at outcome expectancies and/or beliefs about risks, thus counteracting overestimated stocking outcomes and making possible risks more salient in anglers’ minds. This should then impact the anglers’ attitudes toward stocking and, finally, the frequency of their involvement in stocking-related behavior; this is what the significant indirect paths from risk beliefs and outcome expectancies on stocking behavior suggest.

Overall, these are encouraging results that demand further analyses of angler beliefs about stocking to fully understand why and under which conditions anglers favor or disfavor stocking as a management tool. The results regarding their attitudes hint that the state of the environment (degraded vs. near-natural) plays a key role in the anglers’ belief systems regarding stocking. However, this has yet to be analyzed in depth and is a task for future research.

Besides these insights regarding anglers and stocking, there are also some limitations to this study that have to be taken into account when interpreting and deriving implications from the results. First, with a response rate of 25%, the results of the present study can hardly be generalized to the whole population of Swiss anglers. This is because the anglers who responded to the survey might have been the only ones interested in the topic. This means that the respondents might differ systematically from the average Swiss angler concerning experiences, beliefs, and attitudes toward stocking. Unfortunately, we have not controlled for this, and thus, the results should be interpreted cautiously. On the other hand, a sample size of N = 349 can be considered as acceptably high, and the interpretation of both descriptive results and the results of the SEM draw a consistent and meaningful picture. Additionally, the socio-demographics did not indicate any significant bias in the sample when compared to a former study of Swiss anglers. Furthermore, even if only a certain, special group of anglers responded to our survey, the data indicates that it is a group consisting of individuals who are actively involved in management decisions and related work. Thus, these people are likely to be the target group for an intervention with the aim to promote pro-ecological management and abandon inadequate ecosystem management practices.

A second limitation concerns the causality that our analysis and results suggest. We are aware that the associations found in the SEM cannot be interpreted in a strict causal sense. However, where necessary, the item wording clearly referred to the past, present, or future. A further source of criticism may be the sole assessment of self-reported data. We are aware of the ongoing discussion about the validity of self-reported data (e.g., [51], [52]); in the case of our study, the self-reported past behavior used as a proximal indicator for future stocking behavior could be biased by, for example, social desirability. Engaging in voluntary work, such as helping out with stocking-related activities, is highly appreciated in Swiss organizations like fishing clubs. Additionally, the quite high values for acknowledging the normative influence of peers on anglers’ own opinions suggest that social desirability could be an existing motive in our sample. Thus, it might be that our respondents mentioned a slightly higher frequency of involvement in those stocking-related activities. Although we cannot determine how strongly the results of the present study are influenced by social desirability, we argue that the results draw a consistent and meaningful picture of how Swiss anglers think about stocking and what influences them to engage in stocking-related activities.

## Conclusion

The results of the present study nicely depict which factors influence the anglers’ intention to engage in, and actually participate in, stocking activities. These factors include beliefs about stocking-related risks, outcome expectancies, attitudes toward stocking, and perceived behavioral control, whereas normative influence by peers had only a negligible influence on intention. Our analysis also shows how these factors are related to each other, and thus how they contribute to a deeper understanding of why, and under which conditions, anglers favor stocking. The results illustrate the striking role of beliefs about stocking (e.g., risks associated with stocking and outcome expectancies) in the anglers’ attitudes toward stocking and their participation in stocking. These findings should encourage environmentalists and ecosystem managers, when they are planning to abandon inadequate ecosystem management practices like stocking, to focus on those factors that contributed significantly to the formation of stocking intention. Given the discussed risks associated with stocking, an intervention could be designed with the aim of promoting pro-ecological fisheries management. Such an intervention can target outcome expectancies and risk awareness concerning stocking by, for example, conducting stocking success controls in which anglers are involved and can therefore directly perceive whether the stocking measures are successful or not. Through this, the involved anglers would gather new experiences that might influence their beliefs about stocking and that consequently impact their attitudes and intention to engage in stocking. Thus, such an intervention could contribute to abandoning inadequate ecosystem management practices in the long run.

This study further contributes to general understanding of management practices. Although we conducted our research in the domain of recreational inland fisheries, there might be similar structures or problems/controversies in other domains. Therefore, it might be worth transferring our findings to those fields where groups of stakeholders are powerful and dominate the process of management decisions in spite of conflicting scientific evidence.

## Supporting Information

S1 Data Subset **Data file (.txt) containing raw data and variables used for the present publication.**
(TXT)
